# Identification of genotypes of Influenza A virus in Malaysia

**DOI:** 10.12669/pjms.305.5224

**Published:** 2014

**Authors:** Rahman MM, Wong KK, Isahak I, Rashid ZZ, Alfizah H

**Affiliations:** 1 Rahman MM, Professor, Dept. of Medical Microbiology & Immunology, Faculty of Medicine, UKM, Cheras 56000, Kuala Lumpur, Malaysia.; 2 Wong KK, Scholar, Dept. of Medical Microbiology & Immunology, Faculty of Medicine, UKM, Cheras 56000, Kuala Lumpur, Malaysia.; 3 Ilina Isahak, Professor, Faculty of Medicine and Health Sciences, University Sains Islam, Kuala Lumpur, Malaysia.; 4 Rashid ZZ, Senior Lecturer, Dept. of Medical Microbiology & Immunology, Faculty of Medicine, UKM, Cheras 56000, Kuala Lumpur, Malaysia.; 5 Alfizah H, Senior Lecturer, Dept. of Medical Microbiology & Immunology, Faculty of Medicine, UKM, Cheras 56000, Kuala Lumpur, Malaysia.

**Keywords:** Influenza A, rRT-PCR, H1N1, H3N2, H5N1, Phylogenetic analysis

## Abstract

***Objective:*** Influenza is considered as an emerging disease until today. The present study was undertaken to determine the prevalent genotypes of Influenza A virus in Malaysia.

***Methods:*** Influenza A virus was identified from respiratory specimens by real-time reverse transcriptase polymerase chain reaction (rRT-PCR). Phylogenetic analysis of the identified isolates was performed and genotypes were detected.

***Results: ***A total number of 505 throat swabs and nasopharyngeal aspirates were examined by rRT-PCR at Universiti Kebangsaan Malaysia Medical Centre (UKMMC) in which 65(12.87%) were positive for influenza A. The identified isolates were successfully genotyped by phylogenetic analysis. The identified influenza A genotypes were: H1N1 (42), H3N2 (20) and H5N1 (3).

***Conclusion:*** The findings indicated that 3 genotypes were circulating in Malaysia during 2011 in which H1N1 was the predominant. Results added new genotype (H5N1) identification record in Malaysia that may be added in data base of WHO and CDC.

## INTRODUCTION

Influenza virus causes millions of illness associated with respiratory syndromes and approximately 500,000 deaths each year.^1^ Globally, about 20% of children and 5% of adults develop symptomatic influenza each year.^2^

In tropical countries like Malaysia, influenza occurs throughout the year with peak viral activity during the dry season April to June, and the wet season October to January.^3^ Seasonal outbreaks of influenza occur almost round the year. Between 2003 until 2005, influenza outbreaks have been documented from West Malaysia involving mainly residential schools.^4^

From 2005 to 2009, the Institute for Medical Research (IMR), Kuala Lumpur, Malaysia received a total of 7,117 respiratory specimens from patients with influenza-like illness (ILI) for influenza screening. Seasonal influenza virus was isolated from 17.3% of patients with ILI in 2005, 31.6% in 2006, 12.8% in 2007, 10.2% in 2008 and 13.5% in 2009. The study indicated that one or more seasonal influenza A and B virus strains circulating in Malaysia throughout the year. Viral genotypes play an important role in clinical symptoms, however no systematic study on influenza viruses have been conducted yet in Malaysia to find out the genotypes and their relationship with clinical manifestations’. Therefore, the present study was undertaken keeping the above in view.

## METHODS

***Study population & specimens: ***A total of 505 throat swabs and nasopharyngeal aspirates were collected from the patients with respiratory illness during March to August during 2011 at Universiti Kebangsaan Malaysia Medical Centre (UKMMC). These were sent to the laboratory of the Department of Medical Microbiology and Immunology for diagnosis. The patients’ information including age, gender and clinical diagnosis were recorded for analysis. All specimens were immediately tested or kept at 2-4°C (≤72 hours) or frozen at –70^o^C until tested.

***Ethical approval: ***This study was approved by the Research and Ethics Committee, UKMMC (FF-320-2011).

***RNA Extraction: ***It **was** performed as per the method followed by Ken et al.(2012)^5^, briefly 200µl of each sample (nasopharyngeal aspirates or throat swabs) was added to 400µl lysis/binding buffer, containing a chaotropic salt, poly(A) and proteinase K and then incubated for 45 minutes. After centrifugation inhibitor removal buffer was added. Finally purified nucleic acid was eluted in 50µl of elution buffer (High Pure Viral RNA Kit, Roche, Germany).

**Table-I:** Primers and probes used for detection of influenza A virus

***Primer***	***Sequence***	***Gene Target***	***Location (BP)***	***Genbank***
Forward primer	5’-GACCRATCCTGTCACCTCTGAC-3’	M	156-177	HM590431
Reverse primer	5’- GGGCATTYTGGACAAAKCGTCTACG-3’	M	226-250	HM590431
Probe	5’-6FAM-TGCAGTCCTCGCTCACTGGGCACG--BBQ -3’	M	201-224	HM590431

***Real-Time Reverse Transcription Polymerase Chain Reaction (rRT-PCR): ***The method of Ken et al.(2012)^5^ was used with the designed primer (Table-I). An amount of 5µl of purified viral RNA was added to a reaction mixture 15µl containing 0.4µl enzyme blend 50-fold concentration, 4µl reaction buffer 5-fold concentrations, 3µl primers and probe mix and 7.6µl water. A single negative control consisting of water as no template and a single positive control consisting of plasmid control were included for each set of the rRT-PCR reactions.

***Nucleotide Sequencing: ***The purified rRT-PCR products that obtained after amplification of M gene, were sequenced by the automated sequence analyzers CEQ™ 8000 Genetic Analysis System, Beckman Coulter with Genome Lab™ Dye Terminator Cycle Sequencing (Quick Start Kit). The nucleotide sequence data (base-call) produced by automated sequence analyzers CEQ™ 8000 Genetic Analysis System were compared with influenza virus sequences in Genbank to define the genotypes of strain obtain from the study.

***Phylogenetic Analysis: ***Phylogenetic analysis was performed to each virus sequence using software MEGA 5 with Neighbour joining method to create a dendrogram for cluster analysis.

***Classification of Clinical features of the identified influenza A genotypes: ***Clinical characteristics of respiratory virus infected patients were classified into Mild, Moderate and Severe. Mild is considered in case of patients suffered from fever, cough and influenza like illness. Moderate is considered the patients suffered from aspiration pneumonia, atypical pneumonia, bronchopneumonia, viral pneumonia and community acquired pneumonia. Severe is considered the patients suffered from acute bronchiolitis, acute exacerbation of bronchial asthma, chronic lung disease and chronic obstructive airway disease (COAD).

***Statistical Analysis: ***Data analysis was done using SPSS 15.0. Analysis was performed using Chi-square test for qualitative data and student t-test for qualitative data. P value less than 0.05 is considered significant.

***Nucleotide sequence accession number: ***Nucleotide sequence accession numbers obtained for this study are: CY124826.1, CY01445.1 and JQ247216.1.

## RESULTS

**Fig.1:**
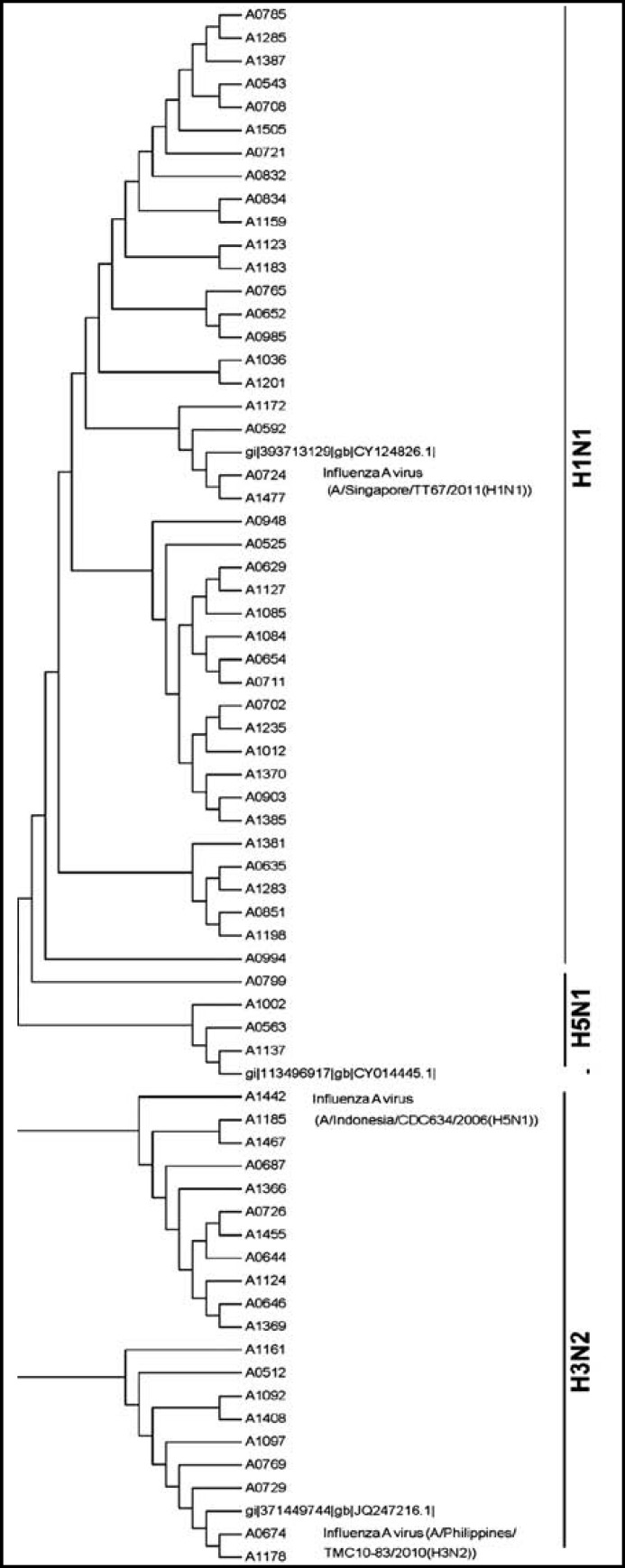
Phylogenetic tree for influenza A nucleotide sequences contructed by using MEGA 5.O neighbor-joining phylogenetic tree. Reference Genbank sequences of strains included to compare the strains. Distribution of specimens selected for analysis by the LC-rRT-PCR. The majority of the isolated virus cluster with the H1N1 genotype

Out of 505 respiratory specimens analyzed during March 2011 to August 2011, 65 were positive for influenza A virus. All 65 positive samples identified were sequenced and phylogenetic analysis was performed (Fig.1). In the study most of the influenza A virus infected patients (67.69%) were less than 3 years old. Considering the gender, 53.38% patients were male.

The 65 influenza A isolates were placed under 3 clusters in phylogenetic tree with the neighbor joining method. Forty two isolates were placed under genotype H1N1, 20 isolates were genotype H3N1 and 3 isolates were under genotype H5N1 (Fig.1). It may be mentioned that genotypes H1N1 and H5N1 formed their own branch and genotypes H3N2 made separate branch. The phylogenetic tree and alignment analysis were performed using Crustal X (Fig.1)*. *Clinical features of the identified genotypes have been tabulated (Table-II) as per the classification of the features.

**Table-II:** Comparison of clinical features of influenza A virus genotypes

***Parameter***	***Variable***	***No. (%) patients (n=65)***			***P value***
		***H1N1***	***H3N2***	***H5N1***	
Clinical symptoms	Mild	14 (21.54%)	6 (9.23%)	1 (1.54%)	
	Moderate	23 (35.38%)	10 (15.38%)	2 (3.08%)	0.865
	Severe	5 (7.69%)	4 (1.54%)	0 (0.00%)	

## DISCUSSION

The study detected 65(12.87%) influenza A virus from respiratory specimens at UKMMC, Malaysia. Phylogenetic analysis revealed that all 65 influenza positive specimens were placed under 3 clusters in phylogenetic tree with the neighbor joining method. **Forty two** isolates were placed under genotype H1N1, 20 isolates were genotype H3N1 and 3 isolates were under genotype H5N1. In molecular epidemiology it is very important to know genotype that would give the source of origin, transmission and migration history. It has been confirmed from the present study that the isolates were from the single influenza outbreak and their genetic relationship was similar to previous outbreaks.

Clinical manifestations of influenza virus A genotype infection were recorded from the patients information sheet. It was observed that genotype H1N1 expressed clinical manifestations of mild (14) moderate (23) and severe type (5).^5^ H3N2 also expressed mild (6) moderate (10) and severe type of manifestations(4). However, genotype H5N1 showed mild (1) and moderate (2) clinical manifestations only. Statistically, association between genotype and clinical manifestation were not found to be significant (p>0.05). Our study indicated that multiple genotype co-circulate in a single epidemic area. The findings are similar to the report of CDC Weekly Flu View^6^ which showed that H1N1 and H3N2 were the predominantly circulating genotypes.

In Malaysia, influenza viruses were found to circulate throughout the year with higher occurrences during the middle part of the year and there were usually 3 to 6 influenza virus genotypes co-circulating simultaneously each year.^3^ In 2000, influenza A (H3N2) emerged in Malaysia and in 2001, influenza A (H1N1) was predominantly circulating strain in Malaysia.^6^ The pattern of circulating influenza viruses isolated mirrored that of the southern hemisphere from March to September 2001, influenza A (H1N1) viruses continued to be responsible for most outbreaks.^7^

In 1997, influenza A viruses of the H1N1 subtype were isolated from patients in the Hong Kong area.^8^^,^^9^ Influenza A and B are the major causative agents of human acute respiratory disease worldwide. Most influenza pandemics are associated with type A influenza virus. Usually, H1N1 and H3N2 are the most common subtypes of influenza A virus found to infect humans.^9^

The most interesting finding for the study was the identification of strain influenza A H5N1. Avian influenza A(H5N1) virus occurs mainly in birds, is highly contagious among birds, and can be deadly, especially in domestic poultry. Avian influenza A(H5N1) virus infections resulting in high mortality in poultry and wild birds have been detected in Asia, the Middle East, Europe, and Africa since December of 2003. Avian influenza A(H5N1) virus infections among domestic poultry have become common (endemic) in certain areas. As of 2011, the United Nations Food and Agriculture Organization considers six countries to be endemic for avian influenza A(H5N1) virus in poultry (Bangladesh, China, Egypt, India, Indonesia, Vietnam).^10^^-^^12^

Based on our data it has been suspected that the human pathogenic avian influenza (HPAI) A (H5N1) may be prevalent in Malaysia too, but the severity of the infection and the area of patients residence and history of infection are yet to find out. However, considering that transmission of influenza A, H5N1 is mainly respiratory route and it might have come from poultry source. Further investigations may need to trace the source of infection.

The present study reaffirmed that rRT-PCR assay was able to detect subtypes of influenza A that included H1N1, H3N2 and H5N1. Detection of H5N1 in Malaysia creates awareness about the possibility of the prevalence of deadly virus avian influenza in Malaysia.
